# Detecting the symptoms of Parkinson’s disease with non-standard video

**DOI:** 10.1186/s12984-024-01362-5

**Published:** 2024-05-03

**Authors:** Joseph Mifsud, Kyle R. Embry, Rebecca Macaluso, Luca Lonini, R. James Cotton, Tanya Simuni, Arun Jayaraman

**Affiliations:** 1 Max Näder Center for Rehabilitation Technologies and Outcomes Research, Shirley Ryan AbilityLab, Chicago, IL USA; 2 Northwestern University, Chicago, IL USA; 3 Center for Bionic Medicine, Shirley Ryan AbilityLab, Chicago, IL USA

## Abstract

**Background:**

Neurodegenerative diseases, such as Parkinson’s disease (PD), necessitate frequent clinical visits and monitoring to identify changes in motor symptoms and provide appropriate care. By applying machine learning techniques to video data, automated video analysis has emerged as a promising approach to track and analyze motor symptoms, which could facilitate more timely intervention. However, existing solutions often rely on specialized equipment and recording procedures, which limits their usability in unstructured settings like the home. In this study, we developed a method to detect PD symptoms from unstructured videos of clinical assessments, without the need for specialized equipment or recording procedures.

**Methods:**

Twenty-eight individuals with Parkinson’s disease completed a video-recorded motor examination that included the finger-to-nose and hand pronation-supination tasks. Clinical staff provided ground truth scores for the level of Parkinsonian symptoms present. For each video, we used a pre-existing model called PIXIE to measure the location of several joints on the person’s body and quantify how they were moving. Features derived from the joint angles and trajectories, designed to be robust to recording angle, were then used to train two types of machine-learning classifiers (random forests and support vector machines) to detect the presence of PD symptoms.

**Results:**

The support vector machine trained on the finger-to-nose task had an F1 score of 0.93 while the random forest trained on the same task yielded an F1 score of 0.85. The support vector machine and random forest trained on the hand pronation-supination task had F1 scores of 0.20 and 0.33, respectively.

**Conclusion:**

These results demonstrate the feasibility of developing video analysis tools to track motor symptoms across variable perspectives. These tools do not work equally well for all tasks, however. This technology has the potential to overcome barriers to access for many individuals with degenerative neurological diseases like PD, providing them with a more convenient and timely method to monitor symptom progression, without requiring a structured video recording procedure. Ultimately, more frequent and objective home assessments of motor function could enable more precise telehealth optimization of interventions to improve clinical outcomes inside and outside of the clinic.

## Introduction

Markerless human pose estimation (HPE) is a powerful technique that has the potential to advance the fields of physical medicine and rehabilitation [1]. The aim of HPE technology is to measure human body kinematics by tracking anatomical keypoints in video data, eliminating the need for traditional motion capture systems [2]. The availability of intuitive and quantitative data through HPE algorithms, combined with expert clinical insight and labeling, opens the door to training supervised machine learning (ML) algorithms. These ML algorithms can then make perceptive inferences about a person’s current medical condition directly from video data. This combination of technologies, referred to as automated video analysis, holds promise across various stages of healthcare, including child developmental tracking [3, 4], adult injury prevention [5], and clinical examinations [6].

Despite its potential, the adoption of automated video analysis technology remains limited among end users [1]. This is partly due to certain limitations in HPE that hinder its practicality, especially for home use in clinical populations. HPE may struggle to accurately track fast and complex movements where there is motion blur or for clinical populations not included in the algorithm’s training dataset [2, 7]. Additionally, HPE algorithms may face challenges in identifying and tracking keypoints for multiple individuals, which is a common scenario in clinical settings involving caregivers and clinicians [8]. Furthermore, the spatial accuracy of HPE algorithms might be insufficient to capture the subtle movements crucial for clinical assessments [9]. Moreover, many existing automated video analysis approaches require a certain level of expertise, which acts as a barrier to entry for both patients and clinicians [10].

In recent years, automated video analysis for symptom tracking in Parkinson’s disease (PD) has gained significant attention [11]. Motor symptoms, such as tremor, bradykinesia, and stiffness, are often the initial indicators of PD onset [12]. Since PD is a progressive neurological disease, regular tracking of symptom progression is crucial to provide optimal treatment. This is also necessary as symptoms can fluctutate throughout the day, such as in response to medication timing. Traditionally, disease progression is monitored through journaling and periodic motor examinations conducted by trained neurologists. However, studies have revealed inaccuracies in journaling, and access to frequent neurologist visits can be challenging for many individuals [13].

Unfortunately, despite the interest in automated video analysis for PD symptoms, most of this technology remains confined to laboratory settings [11]. One reason for this is that certain methods for automated rating of Parkinson’s symptoms require specialized equipment like an RGB-depth camera [14] or wearable sensors [13]. Even methods utilizing only video data often necessitate specific camera setups, including restrictions like multiple camera views with unobstructed backgrounds [15] or consistent camera angles throughout recordings [16, 17]. These constraints impede the translation of this technology to community-level applications where data collection naturally varies in perspective and background.

In this paper, we present an automated video analysis method specifically designed to classify PD symptoms using realistic and varied video data. The objective is to evaluate how well state-of-the-art computer vision techniques can handle unstructured video data and compare the performance to PD detection models built from more structured and controlled data.

## Methods

### Data source

The video data analyzed in this study was obtained as part of the “Clinician Input Study on Parkinson Disease,” [18, 19] a larger research project supported by the Michael J. Fox Foundation. Written informed consent was obtained from all participants for the procedures and sub-analyses conducted. The Institutional Review Board of Northwestern University (Chicago, IL, USA) approved all aspects of this study.

For this protocol, 28 individuals diagnosed with Parkinson’s disease were recruited to participate in a multi-center study. The study sites were located at Northwestern Memorial Hospital (Chicago, IL), Strong Memorial Hospital (Rochester, NY), University of Alabama Hospital (Birmingham, AL), and the University of Cincinnati Medical Center (Cincinnati, OH). The demographic information of the participants is provided in Table 1.

**Table 1:** Demographic characteristics of participants (n=28)

Metric		Mean ± SD
		N (%)
Age (y)	Mean	63.36 ± 9.53
Gender	Male	21 (75%)
	Female	7 (25%)
Race	White	24 (86%)
	Black	1 (3%)
	Asian	1 (3%)
	Not reported	2 (7%)
Ethnicity	Not Hispanic	27 (96%)
	Not reported	1 (3%)
Site	Birmingham	4 (18%)
	Chicago	19 (68%)
	Cincinnati	3 (11%)
	Rochester	1 (3%)

Prior to the experimental protocol, all participants underwent a 12-h period without taking their PD medication, known as the OFF-medication state. Once in the OFF-medication state, participants were asked to perform a Standard Motor Assessment (SMA), which included finger-to-nose, hand pronation-supination, and other motor tasks [20]. As participants performed each task, clinicians observed the severity of tremor, bradykinesia, and dyskinesia to provide a rating of overall disease severity (SMA Overall score). The SMA rating scale ranged from zero to four, with zero indicating no symptoms and four indicating severe symptoms. Following the initial assessment, participants took their medication and subsequently repeated the SMA five more times at 30-min intervals, resulting in a total of six assessments. These time points were chosen to capture motor symptoms at different levels of impairment as the medication took effect.

**Fig. 1:**
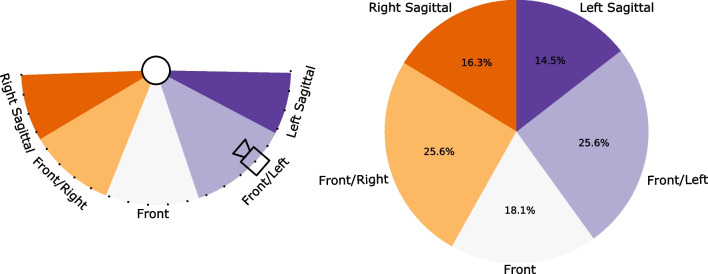
Left: videos were recorded from five perspectives capturing different views of the participant. These perspectives capture the frontal plane, the sagittal plane, and views in between. Perspective did not change for a participant across assessment tasks. Right: the distribution of video perspectives in the data set

Video recordings were captured during each assessment using commercially available handheld smartphones. Since video recording was not the primary focus of the original study, videographers were asked to “film the assessment”, without specific instructions regarding distance, angle, orientation, etc. As a result, videos were captured manually, without the use of a tripod, and from various perspectives (see Fig. 1). Further, the experiment did not mandate or specify a standardized video camera for recording. The frame rate of all videos was consistent at 30 fps, but the video resolution varied among the following: 854 × 480, 960 × 540, 1280 × 720, and 1920 × 1080. These recording conditions realistically replicate scenarios encountered by a digital screening tool or tracking-at-home application

**Fig. 2:**
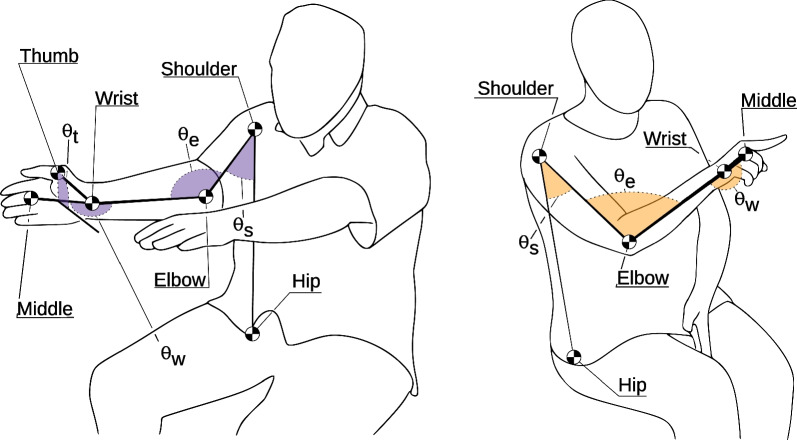
An illustration of the HPS (left) and FTN (right) tasks with labeled keypoints and joint angles. The keypoints tracked during each task are named after the corresponding anatomical landmarks, and $\theta _s$, $\theta _e$, $\theta _w$ and $\theta _t$ the are the angles of the shoulder, elbow, wrist, and thumb joint, respectively

Two tasks from the SMA, namely the finger-to-nose (FTN) and the hand pronation-supination (HPS) tasks, were selected for analysis in this study (see Fig. 2). These tasks were chosen because they can easily be performed and filmed at home. Both tasks require minimal space and can be performed from a seated postion. These advantages lend potential scalability and accessibility to digital screening tools and tracking-at-home applications derived from our approach. During the FTN task, participants were instructed to repeatedly extend one arm at a time to full extension, reaching a target, and then retract their arm to touch their own nose. For the HPS task, participants were asked to extend one hand at a time in front of their body, palm down, and alternately rotate their hand up and down.

### Data processing

**Fig. 3:**
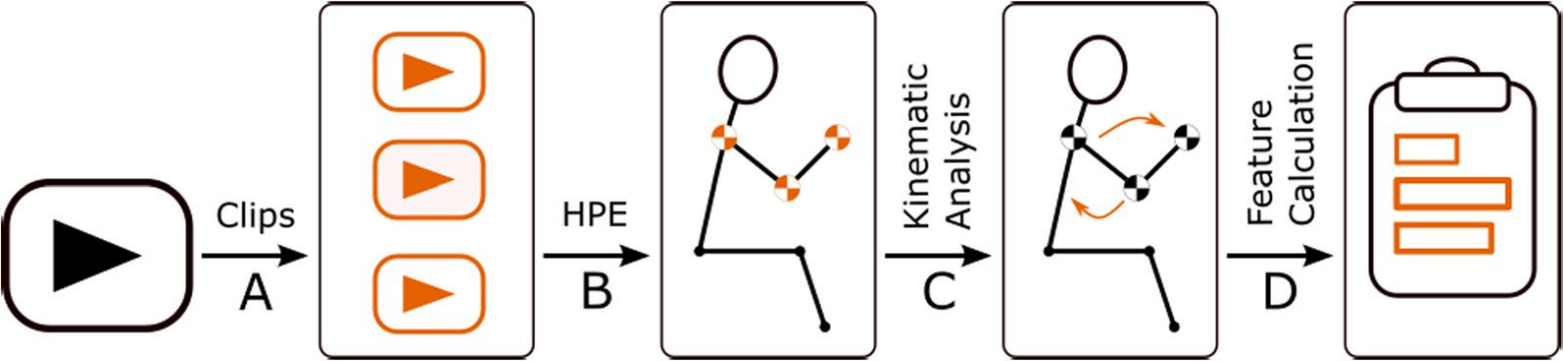
Our data processing procedure has four main steps: **A** manual segmentation of full-length clinical assessment videos into individual tasks; **B** participant keypoints detection using PosePipe and PIXIE to generate timeseries data; **C** calculation of joint kinematics and other timeseries signals from the detected keypoint timeseries; and **D** extraction of advanced features of functional motor performance from the joint kinematics data

Figure 3 provides a high-level overview of the processing steps involved in analyzing the video data and preparing inputs for our machine learning models. To aid with this process, we used PosePipe [21], an open-source tool developed to facilitate markerless HPE pipelines. PosePipe can help with many steps of the analysis pipeline, including video management, manual subject labeling, and data visualization. Initially, we manually segmented the videos obtained during each assessment to isolate the specific task of interest. This resulted in a set of 266 FTN videos and 266 HPS videos collected from 28 individuals. Within all videos, the first step of the analysis pipeline was tracking and manually annotating the person of interest to separate them from other people in the view, such as the assessing clinician. After identifying the patient in each video, we used an HPE algorithm to find the keypoints for only the patient.

#### Pose estimation

We obtained pose estimates from an open-source tool called PIXIE [22] to track the body position of the participant in each video frame. PIXIE estimates the joint angles of the Skinned Multiperson Linear Expressive Model (SMPL-X) model from images [23]. SMPL-X is a widely-used 3D model of the human body (including the hands and face), which was trained using thousands of 3D body scans. Specifically, PIXIE takes every still 2D image in a video as input, and uses a trained neural network to output the parameters of the 3D SMPL-X model that would best describe the image of the person in the frame. These parameters include both the 3D body pose, and a mesh describing the body shape. After the body pose and shape were estimated by PIXIE, we used the forward kinematic model to estimate the location of several 3D keypoints on the human body. The keypoints used for this study are shown in Fig. 2. Each keypoint was transformed into an egocentric frame of defining the ipsalateral (same-side) hip location as the origin of each keypoint’s frame of reference.

#### Kinematic calculations

Multiple joint kinematic signals were derived from the keypoint estimates. For instance, the elbow angle was computed using 3D keypoints from the wrist, elbow, and shoulder joints. All joint angles were determined in 3D space using the positions of three adjacent joint keypoints: the vertex (joint center) and the two neighboring keypoints.

To represent the limb segments around the joint of interest, we created vectors by subtracting the location of the vertex landmark. The angle between these vectors ($\theta$) was then calculated using the formula:

1 $$\cos {\theta } = \frac{\vec {AB} \cdot \vec {CB}}{|\vec {AB}||\vec {CB}|},$$

where (A) is one neighboring keypoint, (B) is the vertex, and (C) is the other neighboring keypoint.

For the FTN data, we calculated three joint angles: the shoulder, elbow, and wrist ($\theta _\text {s}, \theta _\text {e},$ and $\theta _\text {w}$, respectively). The HPS data included one additional keypoint (thumb position) and one additional joint angle: thumb angle ($\theta _\text {t}$). This gives us 8 positional signals for the FTN task (5 keypoints and 3 joint angles), and 10 for the HPS task (6 keypoints and 4 joint angles). Next, we calculated the velocity and acceleration of each keypoint and joint angle using the first and second forward difference numerical derivatives, respectively. This led to 24 kinematics signals for the FTN (8 position, 8 velocity, and 8 acceleration), and 30 kinematic signals for the HPS task (10 position, 10 velocity, and 10 acceleration).

Lastly, we created different filtered versions of each position, velocity, and acceleration signal. We used two different Gaussian filters, with widths of 0.1 s and 0.2 s, to attenuate high-frequency noise from the pose estimation and numerical derivatives. This resulted in a total of 72 signals for the FTN task (24 unfiltered, 24 with filter width 0.1 s, and 24 with filter width 0.2 s), and 90 for the HPS (30 unfiltered, 30 with filter width 0.1 s, and 30 with filter width 0.2 s). Finally, each signal was scaled by its maximum absolute value to obtain a relative signal.

#### Feature calculations

Using the kinematic signals, we developed features for our machine learning algorithm. One common characteristic observed in individuals with bradykinesia, a symptom of Parkinson’s disease, is a reduction in movement speed after multiple repetitions, known as the sequence effect [24, 25]. To measure this effect, we applied a sliding window with a duration of 6.6 s to each kinematic signal and calculated the Fourier transform within each window. Manual observation of the slowest participant showed this value captured at least one repetition of each movement. From the Fourier transform, we were able to extract the most prominent frequency for each window and plot it against time. We then applied a linear regression to the transformed frequency vs. time plot. The coefficients from this regression were utilized as features to train our machine learning models.

**Fig. 4:**
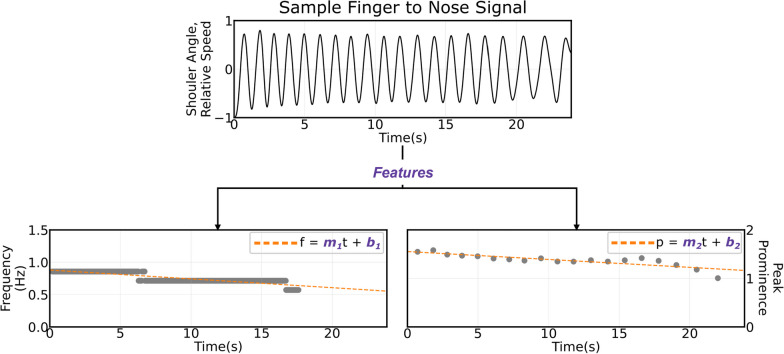
Example of a raw time series signal (top) broken down into its frequency (left) and amplitude (right) features. A sliding window was used to evaluate trends in frequency while peak prominence was used to evaluate trends in amplitude. The coefficients of linear regression were used as features to describe this time series data

Another characteristic commonly observed in individuals with Parkinson’s disease is a decrement of movement amplitude after multiple repetitions [26]. To quantify changes in movement amplitude, we calculated the peak prominence, or how much a maximum value stands out from the surrounding values, for each peak in a kinematic signal. Similar to the frequency analysis, we performed a linear regression and used the regression coefficients as inputs to our models. Figure 4 illustrates how we extracted the features to measure sequence effect and amplitude regression from a sample kinematic signal.

Additionally, we developed a feature to measure pauses between sequences of movements, another characteristic associated with bradykinesia [27]. To quantify these pauses, we introduced a function called the Pause Metric, which calculates the distance below a kinematic threshold for each frame and increments for every frame in the video that falls below the threshold. A higher Pause Metric value indicates longer periods of reduced motion. The Pause Metric is formally defined as:

2 $$P = \sum _t (T - |x_t |)b(|x_t |,T),$$

where (T) is the kinematic threshold, and $x_t$ is the kinematic signal scaled from − 1 to 1. This operation was performed on all of the kinematic signals described in “Kinematic calculations” section (72 for the FTN data, and 90 for HPS). Intuitively, the threshold value *T* can be thought of as a cutoff value, where all scaled kinematic signals ($|x_t |$) below *T* will be considered a “pause,” and the value of the pause metric will be greater the longer the signal remains below *T*. The minimum value of *T* is 0, and the maximum value is 1. Lastly, $b(x_t,T)$ is an expression that determines if the kinematic signal is below the threshold, which we can write as:

3 $$\begin{aligned} b(x_t,T) = {\left\{ \begin{array}{ll} 0 &{} |x_t |\ge T,\\ 1 &{} \text {otherwise}. \end{array}\right. } \end{aligned}$$

Equation 2 is evaluated at every threshold $T \in \{0.05, 0.1,\ldots, 0.95\}$, and a Pause Metric feature was calculated for every video.

To complement our feature set, we combined these custom metrics with descriptive statistics and additional features calculated using the Python package tsfresh [28]. We employed comprehensive extraction settings with the default parameters, resulting in the calculation of over 700 features from each timeseries. Some of these features contained missing values because of signal characteristics like insufficient length or variance.

### Modeling

Since our dataset had less than 13% of SMA Overall scores greater than 1 (see Table 6), we binarized the data, where a score greater than zero was considered the positive class and a score equal to zero was the negative class. After binarizing the data, the FTN task had 55% in the positive class and 45% in the negative class while the HPS task had 77% in the positive class and 23% in the negative class.

We then utilized the previously described features to train two types of binary machine-learning classifiers: a Random Forest (RF) and a Support Vector Machine (SVM). We trained both classifier types (RF and SVM) on both tasks (FTN and HPS), for a total of four classifier algorithms. These types of classifiers have been used for classifying features of Parkinson’s disease in past works [29, 30] and were developed using tools provided by the scikit-learn library in Python [31, 32]. The objective of all four classifiers was to detect whether a participant in the video exhibited symptoms of Parkinson’s disease or not. The ground truth labels for each video were determined based on the SMA overall score, where a score greater than zero indicated the presence of symptoms.

All four classifiers were validated using an 80–20 split with 80% of participants in the training set and 20% of participants in the validation set. We chose to split by participant rather than individual videos to ensure that no videos from the same participant were present in both the training and validation sets. By using this approach, the validation results accurately represented the classifiers’ ability to detect Parkinsonian symptoms in unseen participants. We did not cross-validate across sites. The data in the training sets was used for both model training and hyperparameter tuning.

To optimize our models, we performed hyperparameter tuning using a randomized search that varied parameters such as thresholds for missing features, feature variance, and correlation cutoffs. See Table 2 for a full list and range of all tuned hyperparameters. Once a hyperparameter set was defined by the random search, features that did not meet the threshold for missing data were removed. The remaining features were then transformed by subtracting the mean and scaling to achieve unit variance. Scaled features that did not meet the minimum variance threshold were then removed. For features that were missing data, but met the minimum threshold, gaps were imputed using a nearest neighbors search. Correlated features were then removed and synthetic data was generated by oversampling the minority class using the SMOTE algorithm [33]. This synthetic data was used to bring the minority class up to the approximate sample size of the majority class, which aided with model training. Before validating the parameter set, a LASSO regressor [34] was applied to the features and labels to minimize the remaining features without a strong correlation. The hyperparameter set was then evaluated using a tenfold cross-validation. This process was then repeated a total of 80 times (giving 80 different parameter sets) to identify which set maximized classifier performance, as measured by the F1 score.

**Table 2:** Hyperparameter tuning

Hyperparameter	Value range
Maximum percentage of missing values threshold	[0.0, 1.0)
Minimum variance threshold	[0.0, 0.4)
Maximum correlation coefficient threshold	[0.7, 0.90)
Fill missing, k-neighbors	[1, 4)
LASSO coefficient threshold	[0.01, 0.035)
Synthesize data, k-neighbors	[1, 4)
SVM regularization	[0, 2.0)
SVM $\gamma$	[0, 0.1]

All parameters not described were assigned default values

### Evaluating classifier performance

The performance of each classifier was evaluated with the recall, precision, F1 score, and area under the receiver operating characteristic curve (AUROC). These metrics were calculated from predictions made from videos in the validation set. A high recall indicates that the classifier can accurately identify most participants with symptoms while a high precision assesses the classifiers’ ability to maintain a low false-positive rate. The F1 score balances the benefits of recall and precision to provide an overall assessment of the classifiers’ performance, and a value of 1 would indicate perfect performance. The AUROC measures the classifiers’ overall ability to distinguish instances with symptoms from those without. A score of 0.5 suggests the classifiers performed no better than random guessing, while a score of 1 represents perfect classification.

Further, confidence intervals were estimated by evaluating the classifier on 100 bootstrapped validation sets. Bootstrapped validation sets, with size identical to the validation set, were created by sampling the validation set with replacement. The metrics described above were calculated from the predictions and labels for every bootstrapped set.

## Results

### Model performance

**Fig. 5:**
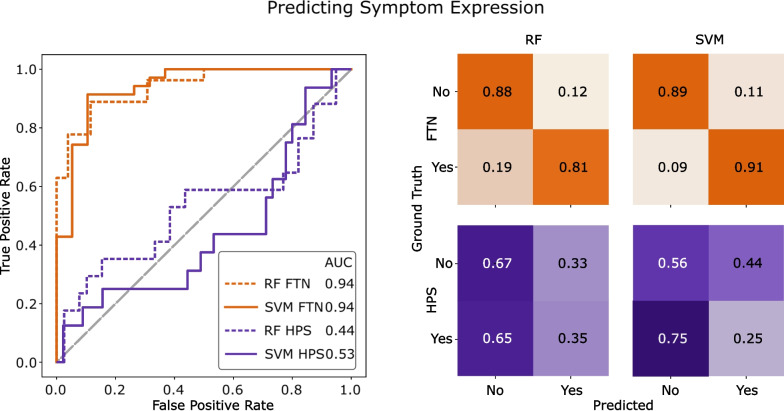
ROC curves with reported AUROC (left) and confusion matrices (right) from the validation set for all combinations of task and model type

Confusion matrices and ROC curves for all models’ predictions on the validation set are illustrated in Fig. 5. When evaluating with the validation set, the AUROC of both the models trained on the FTN task were 0.94, while the AUROC for the SVM and RF models trained on the HPS task were 0.53 and 0.44, respectively. The confusion matrices, evaluated with an operating point of 0.5, also supported the trend of greater performance by models trained on the FTN task. FTN classifiers were able to ability to correctly detect symptoms in 81% (RF) and 91% (SVM) of videos and the absence of symptoms in 88% (RF) and 89% (SVM). In contrast, models trained on the HPS task were only able to correctly detect symptoms in 35% (RF) and 25% (SVM) of videos and the absence of symptoms in 67% (RF) and 56% (SVM). F1 scores for the SVM and RF classifiers evaluated on the validation set were 0.93 and 0.85. Correspondingly, the HPS task yielded lower F1 scores of 0.20 and 0.33 for the SVM and RF, respectively.

Mean values and 95% confidence intervals for precision, recall, and F1 score, for all combinations of task and model type from the bootstrapped sets are presented in Table 3. Among the four models produced, the SVM trained to detect Parkinson’s symptoms using the FTN task yielded the greatest mean F1 score of 0.92, followed by the RF trained on FTN (0.80), RF trained on HPS (0.30), and SVM trained on HPS (0.19). This trend is also present in both precision and recall. Given the gap in performance, all further analysis focuses only on the SVM model trained on the FTN task, unless otherwise specified.

**Table 3:** Mean, precision, recall, and F1 score with 95% confidence intervals from the bootstrapped sets for all combinations of task and model type

Task	Model	Precision	Recall	F1	Videos in test set
		Mean [CI]	Mean [CI]	Mean [CI]	
FTN	SVM	0.93 [0.83, 1.00]	0.92 [0.88, 0.98]	0.92 [0.85, 0.96]	54
FTN	RF	0.81 [0.54, 1.00]	0.82 [0.50, 1.00]	0.80 [0.50, 0.96]	53
HPS	SVM	0.18 [0.00, 0.39]	0.25 [0.00, 0.50]	0.19 [0.00, 0.38]	61
HPS	RF	0.32 [0.00, 0.66]	0.31 [0.00, 0.40]	0.30 [0.00, 0.49]	56

Table 4 outlines the accuracy of our SVM model trained on the FTN task and evaluated on the validation set compared to other binary classifiers focusing on detection of Parkinsonian symptoms. These models were chosen because, together, they broadly cover the current field of automated PD detection. Lonini et al. [18] had subjects complete the SMA, but trained classifiers on data from motion sensors instead of video. The remaining studies all used video data to train their classifiers, but cameras were stationary and activities of interest were finger tapping [35], gait [35, 36], and touching the nose or holding hands in pronation and supination [37]. Since accuracy can be skewed depending on class distribution, we report the positive class for each study as well as a summary of experimental setup.

**Table 4:** Accuracy compared with other methods

Methods	Setup	Class distribution (% positive)	Accuracy
[18]	Motion sensors placed on back of hands	48.5%	0.79^a^
[35]	Stationary Kinect camera (with depth)	90.9%	0.87
[36]	Stationary camera (type not specified)	53.3%	0.81
[37]	Stationary Kinect camera (no depth)	60.0%	0.56
Ours—SVM, FTN	Handheld mobile camera (no depth)	55.3%	0.90

^a^This study reported symptom prevalence by individual data-clips, not by participant

### Selected features

#### Feature types

A total of 13 features were selected to train the SVM model. Table 5 provides details of these features such as the body keypoint and signal each feature was derived from as well as the type of feature (i.e., Fourier coefficient, Pause Metric, regression coefficient, etc.). Of the custom features calculated in “Feature calculations” section, Fourier transform coefficients emerged as the most frequently selected feature type, constituting 4/13 (31%) of the chosen features. Given a median video duration of 19 s, all selected coefficients would fall within the [0.15–0.52) Hz and [3.93–4.23) Hz frequency bands.

The next most common feature types selected were the Pause Metric and regression coefficients, each comprising another 15% of the selected features. The two Pause Metric features were: (1) pauses in the shoulder velocity with a threshold, in dimensionless units, of $T=0.60$, and (2) pauses in the elbow’s angular acceleration with a threshold of $T=0.65$. The two regression coefficient features were: (1) the slope of amplitude vs. time for the elbow angle, and (2) the intercept of frequency vs. time for the finger position. The remaining features were calculated from the tsfresh package.

**Table 5:** Features selected for the SVM classifier

Type	Joint	Signal	Filter width	Additional detail
Fourier coefficient	Shoulder	Position	0.0	0.47–0.52 Hz
	Shoulder	Position	0.0	3.93–3.97 Hz
	Shoulder	Position	0.0	4.19–4.23 Hz
	Shoulder	Velocity	0.0	0.15–0.21 Hz
Pause metric	Shoulder	Velocity	0.1	$T=0.60$
	Elbow	Angular acceleration	0.1	$T=0.65$
Regression coefficent	Elbow	Angle	0.1	Slope of amplitude vs. time
	Finger	Position	0.1	Intercept of frequency vs. time
tsfresh	Wrist	Position	0.0	Mean
	Elbow	Velocity	0.0	Longest strike above mean
	Shoulder	Acceleration	0.0	Aggregate linear trend
	Shoulder	Velocity	0.0	First position of maximum
	Elbow	Velocity	0.0	Sum of reccurring values

After optimizing our models as outlined in “Modeling” section, 13 features were selected for training a binary SVM classifier. The feature type, count, associated joint, signal, filter width, and additional details are all presented. Note that a filter width of 0.0 is simply the unfiltered condition.

#### Feature locations

**Fig. 6:**
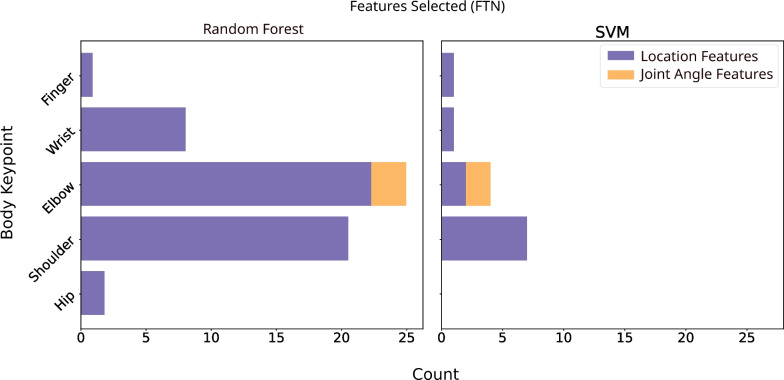
Number of features selected for training SVM model, organized by body keypoint location. Location features from almost all joints were selected while elbow angle was the only joint angle feature selected

As seen in Table 5, 7/13 (54%) of selected features were derived from the kinematic signals of the shoulder joint. This includes all of the Fourier coefficients as well as one of the Pause Metric features. Features derived from the elbow were the next most common with 4/13 (31%), followed by the wrist and finger with 1/13 (8%) each. Additionally, the elbow angle and its angular acceleration were the only angle-related features to be selected. All others were based on the body keypoint’s position, velocity, or acceleration. Each selected feature was associated with one, distinct, filtered signal. When performing a similar analysis on features selected by the RF model, we found the trends in feature location held, including the fact that the only angle-related features selected were derived from the elbow angle (see Fig. 6).

## Discussion

### Performance

Four different machine learning models were evaluated for their ability to detect the presence of Parkinson’s symptoms when trained on kinematics estimated from consumer-grade videos of upper body tasks commonly found in PD clinical assessments. Of these models, the SVM and RF classifiers trained on videos of participants completing an FTN task performed better than their counterparts trained on videos of participants completing an HPS task. All four models were trained on different groupings of participants, which indicates that this discrepancy in performance is likely due to the activity they were trained on. While many of our engineered features (e.g., Pause Metric, frequency features, and peak prominence) were intended to capture symptoms clinicians look for in the HPS task (e.g., halts in movement, sequence effect, decreased movement amplitude), it is possible that these features were more descriptive for the FTN task than the HPS task. Additionally, manual observation of the keypoints revealed that our HPE algorithm (PIXIE, [38]) seemed to track the larger movements of the FTN task more accurately than the smaller hand motions of the HPS task, which may have contributed to the difference in performance. In particular, we observed that individual frames of the hand during HPS were very blurred, making it harder for the HPE tool to consistently identify relevant anatomical landmarks. It is also worth noting that before using the SMOTE algorithm, the FTN dataset had a more equal ratio of symptom presentation (55/45 instead of 77/23). This could also contribute to the relatively lower performance of the HPS algorithm. In the future, further development of descriptive features and deblurring methods [39] may help to improve the accuracy of these models.

Of the models trained on videos of the FTN task, the SVM had greater precision (0.94), recall (0.91), and F1 score (0.93) than the RF (0.88, 0.81, 0.85) while both had the same AUROC (0.94). The performance of the SVM model demonstrates the classifier’s ability to accurately assess the presence or absence of PD symptoms in videos from previously unseen participants. It also meets or exceeds the performance of comparable works that use wearable sensors [18] or video [35–37]. This is promising because, unlike the video-based work we could find, the videos in this dataset were collected from non-standardized perspectives with handheld cameras rather than with a static tripod or other fixed method.

Further, these results are significant because we focused on stationary tasks which can be easily performed in the home and in the absence of the open space typically required for gait analysis which would be useful in the deployment of digital screening tools or tracking-at-home applications. The ability to use common tools in a restricted space lowers the barrier to entry for individuals to easily take these videos at home. Not only that, many of the features we extracted are interpretable, which helps with clinical translation.

### Interpretable features

Healthcare systems are in need of machine learning models that are interpretable [40]. To address this need, we identified and extracted 13 features of functional motor performance which enabled our SVM classifier to yield an F1 score of 0.93. These features included known mathematical calculations such as Fourier transform coefficients as well as custom features engineered to quantify different movement patterns (e.g., sequence effect and pauses in movement). Although the mathematics behind these features may be complicated, the results can provide an intuitive understanding of movement typically associated with symptoms of Parkinson’s disease.

For example, the Fourier coefficients selected by the SVM classifier are on the lower and upper ends of movement frequency of participants’ FTN task in our dataset. These coefficients approximate the amount time spent in the frequency bands from [0.15 to − 0.52) Hz and [3.93–4.23) Hz. Our results showed that these features are predictive of the presence of Parkinsonian symptoms, as scored by the SMA. The consistent framerate cameras onboard consumer-grade smartphones enables precise measurement of the frequency of movements in the frame. Since Fourier coefficients were selected only for the raw signal, and not a filtered signal, it’s probable the Fourier coefficient features from the filtered signals were removed during the pipeline step where correlated features are dropped.

Additionally, the Pause Metric described in Eq. (2) was designed to quantitatively describe a specific symptom of PD: pauses between movements, a characteristic of bradykinesia. The SVM classifier described above took advantage of two Pause Metric features located at the shoulder and elbow. It was determined that the amount of time spent below 60% of a participant’s maximum shoulder speed and the amount of time spent below 65% of a participant’s maximum elbow angular acceleration were useful in determining the presence of symptoms of PD. Unlike black-box techniques, the Pause Metric can be used by clinicians to quantify periods of slower movement, which could lead to a quantitative measure for bradykinesia.

Finally, the sequence effect is a well-known characteristic of movement patterns observed in individuals with PD. Our results confirm that it is both measurable and useful in classifying symptomatic expression, as our model found that changes in frequency of the finger position were helpful in correctly detecting the presence of Parkinson’s symptoms. Just as the Pause Metric can be extracted and tracked over time, changes in movement associated with the sequence effect may also be tracked. This analysis enables quantitative measures of the symptoms of PD and highlights the potential of automated video analysis to bridge the gap from ordinal measures of PD to true continuous measures. Further, these analyses could illuminate new ways to quantify motor symptoms of PD.

Beyond the types of features selected, the locations that selected features were derived from were also intuitive. Both our SVM and RF models relied on features related to the elbow and the shoulder more than other body keypoints. Additionally, both models also used features derived from the kinematics of the elbow angle, but no other joint angle. This result is to be expected as the articulation of this joint is the central component of an FTN task.

An unexpected result, however, was the large number of features selected from shoulder kinematic time series. This may be due to our feature selection process. In an effort to reduce redundant input to our models, we removed features that were highly correlated. It is possible that the predictive information from the shoulder is also present in the elbow, but was removed as the two were highly correlated. Another potential reason for this is that participants may be showing greater compensatory movement at the shoulder to counter decreased (or decreasing) mobility of the elbow. Currently, these compensatory motions may only be identifiable to the highly trained eye of a neurologist. This analysis shows that automated video has the potential to not only reveal these same motions and patterns to an untrained eye, such as a caregiver, but can also provide information about whether or not Parkinson’s symptoms are present. This technique provides the clinician-desired interpretability [41] to any screening tools or tracking-at-home applications derived from it.

### Limitations

The classifiers developed in this work were able to accurately detect the symptoms of PD for many users, but they were not without limitations. The difference in model varied primarily across assessment task. We think this may have been a result of the HPE algorithm selected. Currently, full-body HPE techniques are typically better at tracking gross body movements, like those in the FTN, compared to smaller motions at extremities like the hand [42]. Our results support this statement as tracking a participant’s digits during an HPS task was a challenge for our algorithms.

Prior work has shown that PIXIE is capable of very accurate joint tracking, particularly of the hands. From Table 1 in [38], PIXIE’s error of 11.2/11.0 mm for tracking joints of the left/right hand was lower compared to popular alternatives (12.8/12.4 mm error for left/right hand). This study did not record ground truth keypoint measurements to compare our estimates to, but we believe the blurring we observed during some frames of our videos may have been detrimental to our keypoint tracking accuracy. Computer vision algorithms for video interpolation and deblurring has proven effective for video-based gait analysis [39]. These, combined with approaches that fuse estimates of hand keypoints and SMPL-X parameters [43], could improve performance on this task in the future. However, we feel that the high performance of our PD-symptom detection algorithm is strong evidence that HPE algorithms like PIXIE are able to take accurate enough body measurements to answer clinically-relevant questions, even in the presence of hand blurring. In addition, scaling each signal by its maximum value may further reduce the capability to extract meaningful features from abnormally small movements, which may be important in detecting tremor. Future HPE algorithms can only be expected to improve in performance.

Another limitation is that our current method is not scalable as described. Often, multiple people were in frame during assessments, including: clinicians, the participant, and other researchers. Although the HPE was able to identify the number of people in frame, manual annotation was needed to identify which person was the participant. Deploying a screening tool or tracking-at-home application would require a systematic method to identify people of interest (i.e., patients) to increase usability in a home setting.

Additionally, the supervised learning labels were extracted from the SMA, which is not a widely recognized clinical assessment. All clinicians in the original data collection stage were movement disorder neurologists [19], but few other studies have implemented the SMA. No inter-rater variability study was conducted among our clinical raters, meaning that there may be some unknown biases that vary from rater to rater. Though the two activities chosen for this study are taken from Part III of the MDS-UPDRS, the scoring criteria were not the same and therefore are not directly translatable.

Our results support that the methods described can accurately distinguish between the presence and absence of PD symptoms. In the future, we would like to investigate if this algorithm would also perform well at detecting the onset of new symptoms or progression of existing symptoms over time. This would require a prospective study, in which subjects would be evaluated over a long period of time with both the SMA and our video analysis pipeline, to evaluate if the video method detected the same symptom onset as clinicians.

## Conclusion

Prior work has shown that the ability to detect movement disorders, specifically Parkinson’s disease, would benefit from the development of computer-vision based digital screening tools [11]. In this study, we demonstrated the use of non-standardized video from consumer-grade mobile cameras to detect symptoms of Parkinson’s disease. These videos were recorded from a variety of perspectives on different handheld devices, which at times included camera movement in addition to subject movement. Despite these challenges, which are common occurrences outside of a laboratory setting, this work trained both an SVM and RF classifier to detect symptoms of Parkinson’s disease during an FTN task, which yielded performance that met or exceeded similar methods.

The techniques employed in this study have potential applications in many fields. Any movement disorder characterized by the ability of a person to perform a repetitive movement can be captured by video recorded with consumer-grade cameras. The novel features developed for this study to quantify pauses in movement and the sequence effect could have significant explanatory power for detecting motor symptoms similar to tremor or bradykinesia. Similar digital screening tools or tracking-at-home applications developed with HPE technology may be deployed in the clinical or even the home setting. The outputs they provide can empower both patients and providers with more easily interpretable information to make better decisions when developing a care plan. In the future, HPE techniques and the capabilities of consumer-grade cameras will only advance. These improvements, along with the growing synergy of HPE and ML, will provide the potential to unlock even more digital screening tools for movement disorders.

## Data Availability

The datasets used and/or analysed during the current study are available from the corresponding author on reasonable request.

## Appendix 1: Selected features for FTN models

See Fig. 6.

## Appendix 2: Distribution of SMA overall scores for FTN and HPS

See Table 6.

**Table 6:** Standard motor assessment: overall score distribution

Task	0	1	2	3
FTN	119	115	25	7
HPS	61	127	70	8

## Abbreviations

AUC: Area under curve
AUROC: Area under the receiver operating characteristic curve
FTN: Finger to nose
HPS: Hand pronation/supination
MDS-UPDRS: Movement Disorder Society-Sponsored Revision of the Unified Parkinson’s Disease Rating Scale
HPE: Markerless human pose estimation
ML: Machine learning
SMA: Standard Motor Assessment
SVM: Support vector machine
RF: Random forest
ROC: Receiver operating characteristic
